# Demographics and medical disorders associated with smoking: a population-based study

**DOI:** 10.1186/s12889-020-08858-4

**Published:** 2020-05-15

**Authors:** Wei-Sheng Chung, Pei-Tseng Kung, Hui-Yun Chang, Wen-Chen Tsai

**Affiliations:** 1 Department of Internal Medicine, Taichung Hospital, Ministry of Health and Welfare, Taichung, Taiwan; 2 Department of Health Services Administration, China Medical University, No. 91, Hsueh-Shih Road,, Taichung, 40402 Taiwan; 3 Department of Healthcare Administration, Central Taiwan University of Science and Technology, Taichung, Taiwan; 4 Department of Healthcare Administration, Asia University, Taichung, Taiwan; 5 Department of Medical Research, China Medical University Hospital, China Medical University, Taichung, Taiwan

**Keywords:** Smoking, Sex, Age, Chronic obstructive pulmonary disease, Receiver operating curve

## Abstract

**Background:**

Few studies have investigated factors associated with smoking behaviors. In this population-based study, we investigated demographics and medical comorbid diseases to establish a prediction model for smoking behaviors by using the National Health Interview Survey (NHIS) and National Health Insurance Research Database (NHIRD).

**Methods:**

We enrolled individuals aged ≥40 years who had participated in the NHIS in 2001, 2005, and 2009. We identified the smoking behaviors of the study participants in the NHIS. Smoking behaviors were divided into ever smokers (current smokers and ex-smokers) and nonsmokers (never smokers).We defined medical comorbid disorders of the study participants by using medical claim data from the NHIRD. We used multivariable logistic regression models to calculate the adjusted odds ratio and 95% confidence interval for variables associated with smoking. The significant variables in the multivariable model were included in the receiver operating characteristic curves (ROC) to predict the sensitivity and specificity of the model.

**Results:**

In total, 26,375 participants (12,779 men and 13,596 women) were included in the analysis. The prevalence of smoking was 39.29%. The mean ages of the 16,012 nonsmokers were higher than those of the 10,363 smokers (57.86 ± 12.92 years vs. 53.59 ± 10.82 years). Men outnumbered women among smokers (68.18% vs. 31.82%). Male sex, young age and middle age, being insured categories, residence in suburban areas, and chronic obstructive pulmonary disease (COPD) were independent factors associated with smoking. The area under the ROC curve of these significant factors to predict smoking behaviors was 71.63%.

**Conclusion:**

Sex, age, insured categories, residence in suburban areas, and COPD were associated with smoking in people.

## Introduction

Worldwide, the overall morbidity and mortality in adult smokers are approximately three times higher than those in adult nonsmokers [1]. Smoking leads to diseases and disability in nearly all organs of the body [2]. The major causes of excess mortality among smokers include cancer, pulmonary diseases, and vascular diseases [1]. Hypertension, coronary artery disease (CAD), and stroke are well-established factors of morbidity relating to tobacco smoking [3–5]. Chronic obstructive pulmonary disease (COPD), a heterogeneous disorder causing progressively irreversible airflow limitation, is strongly related to smoking. Smoking accounts for 8 out of 10 COPD-related deaths [6]. The World Health Organization estimated that COPD will become the third leading cause of death by 2030 [7].

Approximately 4 million smokers were present in Taiwan and caused an estimated 18,000 smoke-related death annually [8]. Continued tobacco use results from nicotine addiction, insufficient awareness of risk, and difficulty in abstinence plans, which are driven by diverse psychosocial and socioenvironmental factors, as well as physiological dependence [9]. In older adults, smoking behaviors are more common in men and in those with low education levels, poor health perception, and unmarried status [10].

Information on smoking behaviors is not available in the National Health Insurance Research Database (NHIRD) [11–14]. The evaluation of the effects of smoking on disease development by using the NHIRD is difficult and a couple of epidemiologic studies have listed smoking behaviors as a limitation [15–18]. Therefore, establishing a model to predict smoking behaviors is critical if the researchers do not have access to the study participants. We developed a model that used data on demographics and medical comorbidities from both the National Health Interview Survey (NHIS) and NHIRD to predict smoking behaviors.

## Methods

### Data sources

The Taiwan Ministry of Health and Welfare (formerly Department of Health) has implemented the National Health Interview Survey (NHIS) periodically since 1992 to understand the current status of mental and physical health, health risk behaviors, and medical care utilization. The study participants were national representative samples in the NHIS, which is widely recognized as the most comprehensive and reliable health survey of the civilian, noninstitutionalized, and household population in Taiwan. The Taiwanese government launched a universal National Health Insurance program in Taiwan in 1995, which currently covers more than 99.68% of the country’s residents and is contracted with 97% of healthcare institutions. The National Health Research Institute (NHRI) has created a research data set, NHIRD, containing the claims data of outpatient, inpatient, emergency, and dental care as well as data on prescription drugs dispensed. The NHRI scrambles the identification of the beneficiaries before releasing the NHIRD for public health research. The current study used the NHIS databases of 2001, 2005, and 2009 combined with the NHIRD from 2000 to 2012. Participants younger than 40 years and with incomplete demographics were excluded. We conducted a population-based cohort study and used the diagnoses of medical disorders coded in the International Classification of Disease, Ninth Revision, Clinical Modification (ICD-9-CM), 2001 edition. The Institutional Review Board of authors’ affiliated organization approved this study (CMUH106-REC3–080). The informed consent was waived because of encrypted identification number.

### Definition of outcome variables

We identified the smoking behaviors of the study participants in the NHIS. Smoking behaviors were divided into ever smokers (current smokers and ex-smokers) and nonsmokers (never smokers). Current smokers were individuals who smoked on most or all days, and ex-smokers were individuals who had smoked in the past. The outcome variable was cigarette smoking without any other combustible tobacco product.

### Definition of relevant variables

Data were classified on the basis of sex (male and female) and age (40–64, 65–74, and > 74 years). The insurance categories were category I (employers, employees, and their families in private and public institutions, as well as military personnel), II (occupation union members), III (members of farmers, fishermen and irrigation associations), V (members of low-income households), and VI (veterans and dependents, and unemployed households and their dependents registered in township, city, and district offices). Insured monthly salary categorization of each beneficiary was as follows: ≤17,280 New Taiwan dollars (NTD), 17,280–22,800 NTD, 22,801–28,800 NTD, 28,801–36,300 NTD, 36,301–45,800 NTD, 45,801–57,800 NTD, 57,801–72,800 NTD, and > 72,800 NTD. The considered medical comorbid disorders, defined as the patients being hospitalized once or receiving three or more outpatient diagnoses (principal or secondary) within 365 days of receiving their diagnosis, were hypertension (ICD-9-CM 401–405), stroke (ICD-9-CM 430–438), CAD (ICD-9-CM 410–414), and COPD (ICD-9-CM 491, 492, and 496). The degree of urbanization of residence area where a patient lives was classified into levels, with Level 1 indicating the highest degree of urbanization and Level 7 the lowest.

### Statistical analysis

The distribution of demographic characteristics and comorbidities of ever smokers and nonsmokers was compared. The Chi-square test and two sample Student’s *t* test were used to compare categorical variables and continuous variables, respectively. Furthermore, univariate and multivariable logistic regression models were used to calculate the odds ratio (OR) and 95% confidence interval (CI) for variables associated with ever smokers. The significant variables in the multivariable model were included in the receiver operating curves (ROC) to predict the sensitivity and specificity of the model. The area under the ROC curve represents the efficiency of the prediction model in discriminating between ever smokers and nonsmokers [19]. Data were analyzed and managed using SAS 9.4 (SAS Institute, Inc., Cary, NC, USA). Two-tailed *P* < 0.05 was considered statistically significant.

## Results

### Demographic characteristics and comorbidities of study participants

A total of 26,375 participants—12,779 men and 13,596 women—were included in the analysis. The mean age of the study participants was 56.18 ± 12.31 years. Most participants (53.44%) were aged 40–54 years. Among these study participants, 10,363 people (39.29%) were ever smokers. The majority of the study participants (84.04%) were insured under the category of employers, employees, and their families. Only 1% of the study participants were members of low-income households. Moreover, 47.94% of the study participants resided in suburban areas. The prevalent medical comorbid disorders in the study participants were hypertension (27%), CAD (8.66%), stroke (6.14%), and COPD (5.04%). The prevalence rate of ever smoking accounted for 39.29% of the study participants. Furthermore, 35.55% of the study participants had participated in adult preventive care. (Table 1).

**Table 1:** Demographic characteristics and comorbidities of study participants

**Variables**	**N**	**(%)**	**Variables**	**N**	**(%)**
**Sex**			**Insured category**		
Men	12,779	(48.45)	Categorized I	9883	(37.47)
Women	13,596	(51.55)	Categorized II	5718	(21.68)
**Age (y)**			Categorized III	6566	(24.89)
40–54	14,096	(53.44)	Categorized V	265	(1.00)
55–64	5589	(21.19)	Categorized VI	3943	(14.95)
65–74	3928	(14.89)	**Insured monthly salary (NTD)**		
> 74	2765	(10.48)	≤17,280	1441	(5.46)
**Comorbidity**			17,280–22,800	13,616	(51.62)
**COPD**			22,801–36,300	6150	(23.32)
No	25,045	(94.96)	> 36,301	5168	(19.59)
Yes	1330	(5.04)	**Urbanization of residence areas**		
**Hypertension**			I	5477	(20.77)
No	19,253	(73.00)	II, III	12,107	(45.90)
Yes	7122	(27.00)	IV, V	5761	(21.84)
**Stroke**			VI, VII	3030	(11.49)
No	24,756	(93.86)	**Smoking**		
Yes	1619	(6.14)	No	16,012	(60.71)
**CAD**			Yes	10,363	(39.29)
No	24,091	(91.34)			
Yes	2284	(8.66)			
**Adult preventive care**					
No	16,999	(64.45)			
Yes	9376	(35.55)			

### Demographic characteristics and comorbidities between ever smokers and nonsmokers

Most ever smokers were men (68.18%) and in the age group of 40–64 years (85.06%). By contrast, most nonsmokers were women (64.31%), and 67.89% were 40–64 years. The mean age of nonsmokers were higher than that of ever smokers (57.86 ± 12.92 y vs. 53.59 ± 10.82 y, *P* < 0.001). More ever smokers resided in the suburban areas compared with nonsmokers (47.94% vs. 44.59%). The prevalence of the following medical comorbid disorders was higher in the nonsmokers than in the ever smokers: COPD (5.26% vs. 4.70%), hypertension (29.56% vs. 23.05%), stroke (6.88% vs. 5.00%), and CAD (9.67% vs. 7.09%). More nonsmokers tended to receive adult preventive care than ever smokers (36.51% vs. 34.06%). (Table 2).

**Table 2:** Demographics and comorbidities between ever smokers and nonsmokers

**Variables**	**Total**	**Non-smokers**		**Ever smokers**		*P*-value
		N	(%)	N	(%)	
**Total**	26,375	16,012	(60.71)	10,363	(39.29)	
**Sex**						< 0.001
Men	12,779	5714	(35.69)	7065	(68.18)	
Women	13,596	10,298	(64.31)	3298	(31.82)	
**Age (y)**						< 0.001
40–54	14,096	7785	(48.62)	6311	(60.9)	
55–64	5589	3085	(19.27)	2504	(24.16)	
65–74	3928	3010	(18.80)	918	(8.86)	
> 74	2762	2132	(13.32)	630	(6.08)	
**Mean ± SD**	56.18 ± 12.31	57.86 ± 12.92		53.59 ± 10.82		< 0.001^**§**^
**Insured category**						< 0.001
Category I	9883	5911	(36.92)	3972	(38.33)	
Category II	5718	3212	(20.06)	2506	(24.18)	
Category III	6566	4376	(27.33)	2190	(21.13)	
Category V	265	118	(0.74)	147	(1.42)	
Category VI	3943	2395	(14.96)	1548	(14.94)	
**Insured monthly salary (NTD)**						< 0.001
≤ 17,280	1441	863	(5.39)	578	(5.58)	
17,280–22,800	13,616	8611	(53.78)	5005	(48.30)	
22,801–36,300	6150	3581	(22.36)	2569	(24.79)	
> 36,301	5168	2957	(18.47)	2211	(21.34)	
**Urbanization of residence area**						< 0.001
I	5477	3363	(21.00)	2114	(20.40)	
II, III	12,107	7139	(44.59)	4968	(47.94)	
IV, V	5761	3594	(22.45)	2167	(20.91)	
VI, VII	3030	1916	(11.97)	1114	(10.75)	
**Comorbidity**						
**COPD**						0.040
No	25,045	15,169	(94.74)	9876	(95.3)	
Yes	1330	843	(5.26)	487	(4.70)	
**Hypertension**						< 0.001
No	19,253	11,279	(70.44)	7974	(76.95)	
Yes	7122	4733	(29.56)	2389	(23.05)	
**Stroke**						< 0.001
No	24,756	14,911	(93.12)	9845	(95.00)	
Yes	1619	1101	(6.88)	518	(5.00)	
**CAD**						< 0.001
No	24,091	14,463	(90.33)	9628	(92.91)	
Yes	2284	1549	(9.67)	735	(7.09)	
**Adult preventive care**						< 0.001
No	16,999	10,166	(63.49)	6833	(65.94)	
Yes	9376	5846	(36.51)	3530	(34.06)	

Chi-square test; ^§^Two sample Student’s *t* test.

### Factors associated with ever smokers

**Fig. 1:**
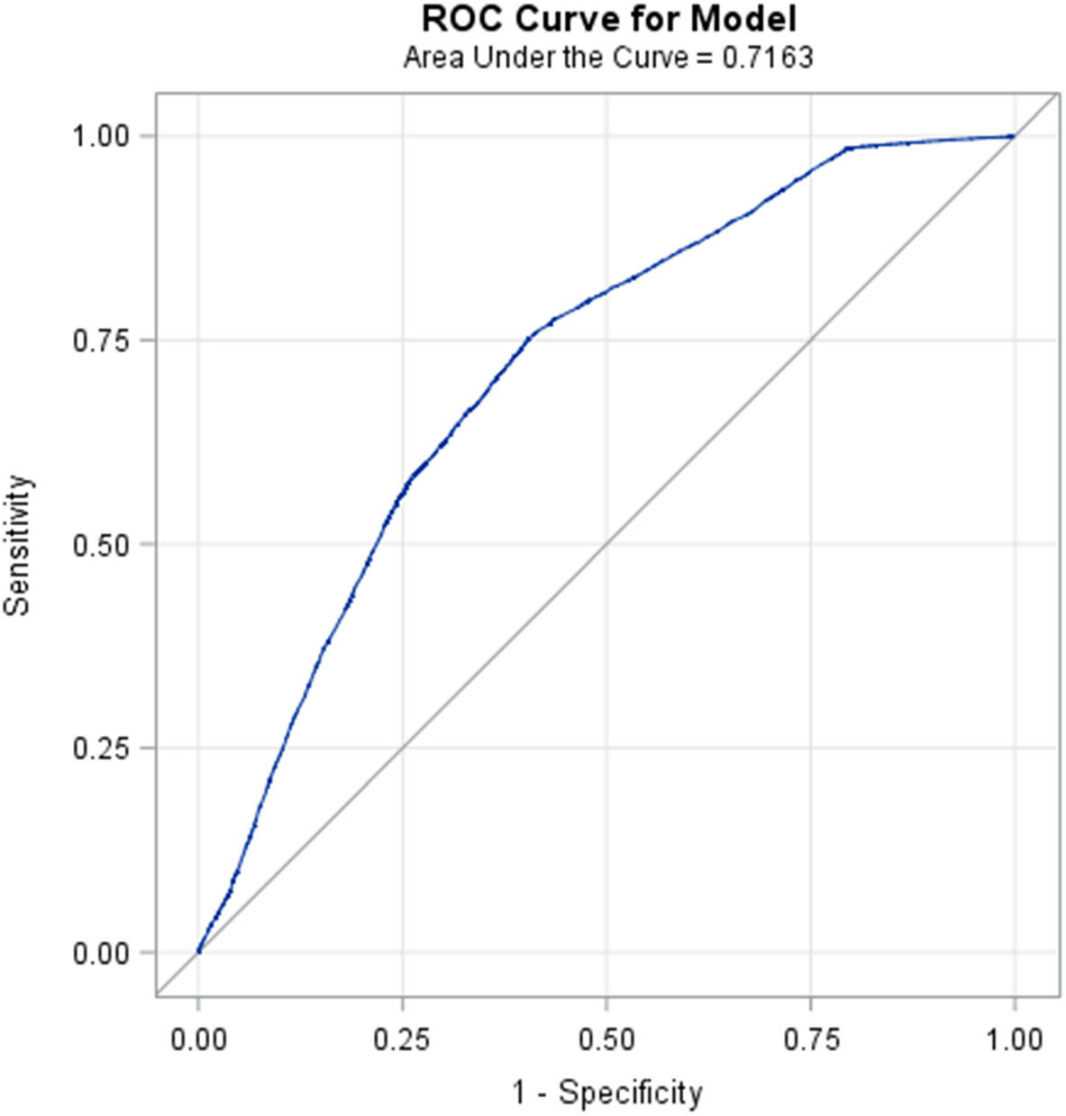
ROC curve of the prediction model for ever smokers

Table 3 lists factors associated with ever smokers by using multivariable logistic regression. Men exhibited a 4.18-fold adjusted OR of ever smoking compared with women (95% CI = 3.96–4.42). Compared with individuals aged > 74 years, those aged 40–54 years and 55–64 years exhibited a 3.12-fold (95% CI = 2.79–3.49) and 3.16-fold (95% CI = 2.82–3.54) adjusted OR of ever smoking. Compared with insured category I, other insured categories exhibited a significant association with ever smoking. Individuals residing in suburban areas exhibited a 1.09-fold adjusted OR of ever smoking compared with those residing in urban areas (95% CI = 1.01–1.17). COPD exhibited a 1.15-fold adjusted OR of ever smoking (95% CI = 1.02–1.31). Furthermore, we incorporated the factors significantly associated with ever smoking into the prediction model; the area under the ROC curve was 71.63%. (Fig. 1).

**Table 3:** Logistic regression model evaluating factors associated with ever smoking

**Variables**	**Unadjusted**				**Adjusted**			
	**OR**	**(95% CI)**		***P*value**	**OR**	**(95% CI)**		***P*value**
**Sex**								
Women	1				1			
Men	3.86	(3.66,	4.07)	< 0.001	4.18	(3.96,	4.42)	< 0.001
**Age (y)**								
> 74	1				1			
40–54	2.74	(2.50,	3.02)	< 0.001	3.12	(2.79,	3.49)	< 0.001
55–64	2.75	(2.48,	3.05)	< 0.001	3.16	(2.82,	3.54)	< 0.001
65–74	1.03	(0.92,	1.16)	0.592	1.09	(0.96,	1.23)	0.176
**Insured Category**								
Category I	1				1			
Category II	1.16	(1.09,	1.24)	< 0.001	1.31	(1.20,	1.43)	< 0.001
Category III	0.75	(0.70,	0.80)	< 0.001	1.18	(1.05,	1.31)	0.004
Category V	1.85	(1.45,	2.37)	< 0.001	2.09	(1.56,	2.80)	< 0.001
Category VI	0.96	(0.89,	1.04)	0.313	1.25	(1.13,	1.38)	< 0.001
**Insured monthly salary (NTD)**								
≤ 17,280	1				1			
17,280–22,800	0.87	(0.78,	0.97)	0.012	0.90	(0.78,	1.04)	0.160
22,801–36,300	1.07	(0.95,	1.20)	0.249	1.11	(0.96,	1.28)	0.149
> 36,301	1.12	(0.99,	1.26)	0.070	0.98	(0.85,	1.13)	0.800
**Urbanization of residence area**								
I	1				1			
II, III	1.11	(1.04,	1.18)	0.002	1.09	(1.01,	1.17)	0.019
IV, V	0.96	(0.89,	1.04)	0.284	1.05	(0.95,	1.15)	0.344
VI, VII	0.93	(0.84,	1.01)	0.095	1.03	(0.92,	1.15)	0.609
**Comorbidity**								
**COPD**								
No	1				1			
Yes	0.89	(0.79,	0.99)	0.041	1.15	(1.02,	1.31)	0.029
**Hypertension**								
No	1				1			
Yes	0.71	(0.67,	0.76)	< 0.001	0.95	(0.89,	1.02)	0.140
**Stroke**								
No	1				1			
Yes	0.71	(0.64,	0.79)	< 0.001	1.02	(0.90,	1.16)	0.730
**CAD**								
No	1				1			
Yes	0.71	(0.65,	0.78)	< 0.001	0.99	(0.88,	1.13)	0.922
**Adult preventive care**								
No	1				1			
Yes	0.90	(0.85,	0.95)	< 0.001	1.05	(0.99,	1.11)	0.116

## Discussion

Smoking leads to disease and disability of nearly every organ of the body [2, 20]. Smoking also remains the leading preventable cause of premature death [21, 22]. The evaluation of the factors associated with smoking behaviors plays a vital role in controlling tobacco use. This is the first study to predict smoking behaviors by using a population-based cohort through a combination of the NHIS database and NHIRD. We observed that sex, age, insured category, residence in suburban areas, and COPD were independent risk factors associated with smoking. Combining these significant risk factors can yield a prediction accuracy rate of 71.63% for people with smoking behaviors.

This study retrieved the database of NHIS in the year 2001, 2005, and 2009, which indicated a smoking prevalence rate of 39.29%. Previous studies have demonstrated approximate smoking prevalence rates of 33 and 22% in Taiwan in 2002 and 2007, respectively [8, 23]. The discrepancy between our finding and those of previous reports may be attributed to the differences in the methodologies. The participants in the current study were aged ≥40 years. Most ever smokers in the current study were men, which is consistent with the finding of previous reports [24–26]. A Global Adult Tobacco Survey in 16 countries revealed that 48.6% of men and 11.3% of women consumed tobacco [26]. In the current study, 55.3% of men and 24.3% of women were ever smokers.

The higher prevalence of comorbidities in nonsmokers than in ever smokers may be attributed the higher mean age of nonsmokers than that of ever smokers. The prevalence of comorbidities such as hypertension, stroke, CAD, and COPD increased with age [27–29]. The increase in blood pressure with age is related to structural changes in the arteries and arterial wall stiffness, which results in the increasing risks of CAD and stroke with age [29, 30].

COPD is characterized by productive cough and dyspnea, a progressive decline in lung function, a deteriorating effect on quality of life, and a high risk of morbidity and early mortality [31]. Environmental toxin exposure, genetic abnormalities, and accelerated aging are risk factors of COPD [32]. However, smoking is identified as the most common risk factor associated with COPD development [31, 32]. In the present study, COPD was significantly associated with smoking after adjustment for covariates.

Certain limitations should be considered while interpreting the study findings. First, the current study provided a correlation rather than a causal connection. Second, the study did not define the dose–response relationship between smoking and associated covariates. Third, despite a meticulous study design with adequate control of covariates, a key limitation of this study is the potential for bias because of possible unmeasured covariates. Fourth, we did not have information to discern the order in which smoking behaviors occurred or when COPD developed among participants. Finally, this study did not include the sample weight in the analyses which may mitigate the representative of nationwide population. However, the strength of our study is that we used a large population-based cohort from the NHIS through random sampling of the nationwide representatives and combined with the medical reimbursement data of the study participants from the NHIRD.

## Conclusions

The present study indicates that sex, age, insured categories, residence in suburban areas, and COPD are significantly associated with smoking behaviors. The prediction model yields a relatively high accuracy in discriminating between ever smokers and never smokers.

## Data Availability

Regarding the data availability, data were obtained from the National Health Interview and Survey and the National Health Insurance Research Database published by the Ministry of Health and Welfare, Taiwan. Due to legal restrictions imposed by the Taiwan government related to the Personal Information Protection Act, the database cannot be made publicly available. All researchers can apply for using the databases to conduct their studies. Requests for data can be sent as a formal proposal to the Health and Welfare Data Science Center of the Ministry of Health and Welfare (http://www.mohw.gov.tw/EN/Ministry/Index.aspx). Any raw data are not allowed to be brought out from the Health and Welfare Data Science Center. The restrictions prohibited the authors from making the minimal data set publicly available.

## Abbreviations

CAD: Coronary artery disease
COPD: Chronic obstructive pulmonary disease
NHIS: National Health Interview Survey
NHI: National health Insurance
NHIRD: National Health Insurance Research Database
NHRI: National Health Research Institute
NC: *North Carolina*
CI: Confidence interval
ROC: Receiver operating curves
ICD-9-CM: International Classification of Diseases, Ninth Revision, Clinical Modification
OR: Odds ratio
NTD: New Taiwan Dollar
